# *In Silico* Theoretical Molecular Modeling for Alzheimer’s Disease: The Nicotine-Curcumin Paradigm in Neuroprotection and Neurotherapy

**DOI:** 10.3390/ijms12010694

**Published:** 2011-01-19

**Authors:** Pradeep Kumar, Viness Pillay, Yahya E. Choonara, Girish Modi, Dinesh Naidoo, Lisa C. du Toit

**Affiliations:** 1 Department of Pharmacy and Pharmacology, University of the Witwatersrand, 7 York Road, Parktown, 2193, Johannesburg, South Africa; E-Mails: pradeep.kumar@students.wits.ac.za (P.K.); yahya.choonara@wits.ac.za (Y.E.C.); lisa.dutoit@wits.ac.za (L.C.d.-T.); 2 Division of Neurosciences, Department of Neurology, University of the Witwatersrand, Johannesburg, South Africa; E-Mail: gmodicns@mweb.co.za; 3 Division of Neurosciences, Department of Neurosurgery, University of Witwatersrand, Johannesburg, South Africa; E-Mail: dineshnaidoo@yahoo.com

**Keywords:** amyloid-β protein, Alzheimer‘s disease, molecular mechanics, artificial neural networks, curcumin, nicotine, isobolographic analysis, docking, central composite design, constraint optimization, ligand-protein complexes, synergism

## Abstract

The aggregation of the amyloid-β-peptide (AβP) into well-ordered fibrils has been considered as the key pathological marker of Alzheimer‘s disease. Molecular attributes related to the specific binding interactions, covalently and non-covalently, of a library of compounds targeting of conformational scaffolds were computed employing static lattice atomistic simulations and array constructions. A combinatorial approach using isobolographic analysis was stochastically modeled employing Artificial Neural Networks and a Design of Experiments approach, namely an orthogonal Face-Centered Central Composite Design for small molecules, such as curcumin and glycosylated nornicotine exhibiting concentration-dependent behavior on modulating AβP aggregation and oligomerization. This work provides a mathematical and *in silico* approach that constitutes a new frontier in providing neuroscientists with a template for *in vitro* and *in vivo* experimentation. In future this could potentially allow neuroscientists to adopt this *in silico* approach for the development of novel therapeutic interventions in the neuroprotection and neurotherapy of Alzheimer‘s disease. In addition, the neuroprotective entities identified in this study may also be valuable in this regard.

## 1. Introduction

*“Neuroscientists are pretty sure they know what causes Alzheimer’s disease, but their theory has not yet given rise to effective drugs.”*—Alison Abbott, The Plaque Plan [1]

Neurodegenerative disorders (NDs) are sporadic and/or familial and characterized by the persistent and progressive loss of neuronal subtypes [2] and includes mainly Alzheimer's disease (degeneration of basal forebrain cholinergic neurons), Parkinson's disease (degeneration of nigro-striatal dopaminergic neurons) and Huntington‘s disease (striatal, hypothalamic and cortical degeneration) [2,3], stroke (necrotic infarcts coupled with inflammatory gliosis), Amyotrophic Lateral Sclerosis (ALS) (upper and lower motor neuronal degeneration and atrophy), multiple sclerosis (lesions and plaques) and HIV-1-associated neurocognitive disorders [2,4]. NDs may impact various brain functions, such as movement (as in Parkinson‘s disease and ALS) or memory and cognition (as in Alzheimer‘s disease). Neuro-regenerative therapies include neuroprotection, nuritogenation and neurorestoration of neuronal subtypes especially with traumatic brain and spinal cord injuries.

**Figure 1:**
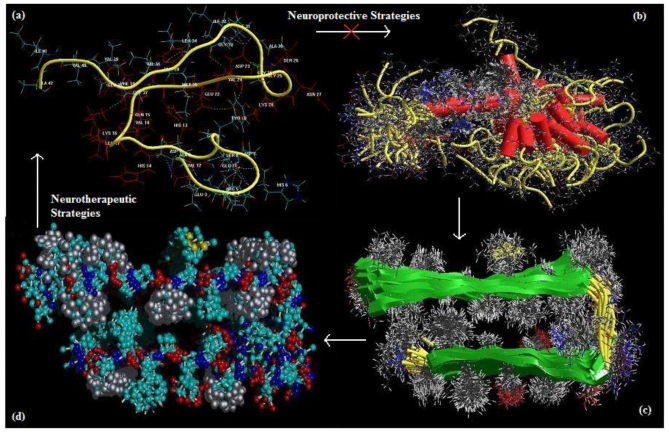
Hierarchical self-assembly of amyloid β-protein (**a**) amyloid-β protein oligomer; (**b**) proto-fibril; (**c**) fibril; and (**d**) plaque deposit.

Alzheimer‘s disease (AD) is a progressive neurodegenerative disorder, encompassing the deterioration of cognitive functions and behavioral changes, characterized by the aggregation of amyloid β-protein (Aβ) into fibrillar amyloid plaques in selected areas of the brain with the lipid-carrier protein apolipoprotein E (apoE), the microtubule associated protein tau, and the presynaptic protein α-synuclein [2,5–7]. High levels of fibrillary Aβ*,* the main constituent of senile plaques, are deposited in the AD brain that results in the loss of synapses, neurons and impairment of neuronal function [8]. Aβ is derived from the amyloid precursor protein through sequential protein cleavage by aspartyl protease, β-secretase and presenilin-dependent β-secretase triggering a cascade of events such as neurotoxicity, oxidative damage, and inflammation that contributes to the progression of AD. Aβ fibrillization involves formation of dimers and small oligomers followed by growth into protofibrils and fibrils via a complex multistep-nucleated polymerization that eventually forms Aβ plaques or deposits (Figure 1) [9].

Apart from Aβ fibrils, smaller species of aggregated Aβ, known as Aβ oligomers, also represent the primary toxic species in AD [10]. Anti-amyloidogenic therapy primarily involves the reduction of Aβ production, inhibiting secretase, increasing Aβ clearance, or blocking Aβ aggregation (with antibodies, peptides, or small organic molecules that selectively bind and inhibit Aβ aggregate and fibril formation) via inhibition of the nucleation-dependent polymerization model [9,11]. Therefore, the use of small molecules and peptides that can induce the Aβ peptide to fold into an α-helical or random, extended chain structure and the detrimental β-sheet structures to form insoluble amyloids may offer a promising alternative to the pharmacotherapy for AD as inhibitors of Aβ oligomerization [12]. Apart from the dose-dependent inhibition of the formation of Aβ fibrils from Aβ40 and Aβ42 and their extensions, destabilization of preformed Aβ fibrils is also an interesting therapeutic intervention [13]. A number of small molecules have been reported to inhibit Aβ fibrillogenesis or to modulate Aβ fibrillization thereby inhibiting Aβ-mediated cellular toxicity resulting from soluble amyloid oligomers or prefibrillar aggregation intermediates [14,15].

Drug discovery, modeling and delivery techniques have benefitted profoundly by the adoption of computational methods that assist in the design of new therapeutic strategies in a more rapid and intricate manner. *In silico* drug modeling that was employed in the present study, encompasses computational methodologies for compound database searching that utilize data from static lattice atomistic simulations of protein-ligand interactions to design a rationalized combinatorial approach for the neuroprotection and neurotherapy of AD based on the molecular interactions of small therapeutic entities with the Aβ-42 monomer. The various steps constituting the current *in silico* modeling process involved: (i) identifying effective neuroprotective entities (NEs) of therapeutic interest based on energy minimizations of the protein-NEs complexes (using Molecular Mechanics Simulations and selection of the most sensitive NEs employing Artificial Neural Networks optimization); (ii) recognizing the site of interaction of the selected NEs on the amyloid protein (using Molecular Mechanics Force Fields and Advanced Docking Techniques); (iii) employing Interactive Data Analysis as a combinatorial approach (using Isobolographic Analysis); and (iv) determining the design of the synergistic combinations and their optimization (via Design of Experiments using a Face-Centered Central Composite Design) to obtain the most stabilized geometrical preferences of the protein-NEs complexes derived from Molecular Mechanics calculations. Ligand- and target structure-based strategies are widely used in virtual screening, but there is currently no methodology available that integrates the extent of the above modeling approaches. In this study, we provide an *in silico* approach that has combined molecular mechanics, stochastic sensitivity analysis, Design of Experiments and interaction studies to design a combinatorial therapeutic strategy for the neuroprotection and neurotherapy of AD.

## 2. Results and Discussion

Aβ, similar to other globular proteins, appear to require essential contribution from both hydrophobic and ionic interactions during structure formation with hydrophobicity providing a large energetic contribution [16,17]. Apart from providing the stabilization energy, these non-bonding interactions provide loose network structures, so that Aβ can tolerate residue replacements at packing positions without losing its stability or shape. Considering these generalized rules of protein structure, it has been assumed that binding of small molecules to a site on Aβ with significant specificity may inhibit amyloid fibril formation and other types of aggregation [18]. Therefore, the mechanism of action of amyloid aggregation inhibitors in terms of blocking oligomer formation, blocking fibril formation, de-aggregating the preformed fibers or rendering Aβ insoluble holds promise for neuroprotection as well as neurotherapy. The chemical disruption of this β-sheet containing polymers was exemplified approximately two decades ago, when the conformational modification of the KLVFF region of Aβ was postulated as a lead for the development of anti-Aβ agents [19]. The following small molecular candidates reported previously to bind amyloid, to modulate protein aggregation and/or toxicity or screened for such activities were modeled in this study for their ability to interact with the Aβ-42 oligomer and included the following: apigenin (APG) [20], congo red (CR) [21,22], curcumin (C) [9,13], dihydroxybenzophenone (DHB) [20], indomethacin (IND) [23], thioflavin T (ThT) [24], hexamethylpyridinium (HMP) [18], glycosylated nornicotine (G) [12], neocuproine (NEO) [25] and polystyrene sulfonate (PSS) [26].

### 2.1. Static Lattice Atomistic Simulations of Protein-NE Complexes to Select Optimal Neuroprotective Entities for Effective Aβ Protein Binding via AMBER Force Field

**Figure 2:**
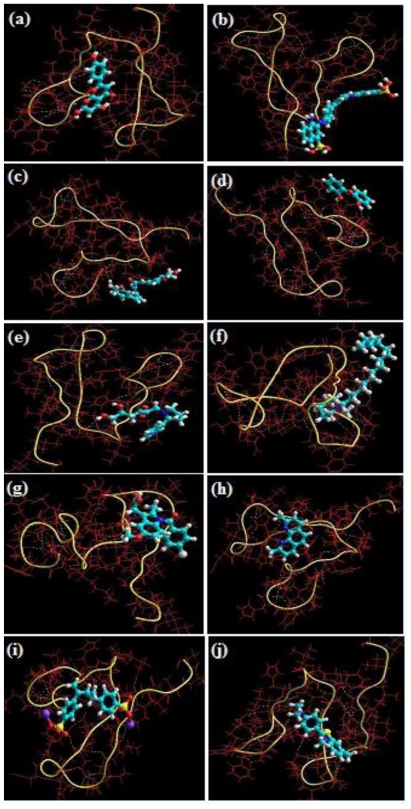
Energy minimized geometrical preferences of the protein-NE complexes derived from Molecular Mechanics computations: (**a**) apigenin; (**b**) congo red; (**c**) curcumin; (**d**) dihydroxybenzophenone; (**e**) glycosylated nornicotine; (**f**) hexadecylmethylpiperidinium; (**g**) indomethacin; (**h**) neocuproine; (**i**) polystyrene sulfonate and (**j**) thioflavin T.

In order to select the best Neuroprotective Entity (NE) for potential neuroprotective or neurotherapeutic activity against Alzheimer‘s disease (AD) or influencing the fibrillization or plaque formation of Aβ-protein, Molecular Mechanics was employed to determine the individual protein-ligand interaction energies between Aβ_1-42_ and 10 NEs in order to understand the structures (Figure 2) and energetics (Table 1) of protein-NE complexes resulting from the interplay between the bonding and non-bonding potentials. This provided the comparative *in silico* profile of the most sensitive NEs of Aβ without undertaking any extensive *in vitro* and *in vivo* studies. Molecular Mechanics described the energies of the complexes in terms of a simple function potentials typically consisting of two sets of terms: one accounted for distortion from ideal bond distances and angles and the other for non-bonded Van der Waals (VdW) and Coulombic interactions, where the bond and angle terms were defined in a self-consistent manner giving an energy minimum at an unstrained structure [27]. The individual energies of the NE molecules were insignificant (as compared to that of the target Aβ) to be considered for the computation of ΔE_interaction_ (E_Host:Guest_ − E_Host_ − E_Guest_). The total steric energy, considered for the interpretation of modeling characteristics, are listed in Table 1. Most of the NEs studied demonstrated high energy stabilized structures (Table 1) and interactions with Aβ in terms of H-bonding (Figure 2).

The molecules are listed in decreasing order of the total minimized energy obtained after MM+ simulations. It is evident from the results that Congo Red and Glyconornicotine constituted the least and most stable geometrical configurations after molecular interaction with Aβ protein (a difference of ≈70 kcal/mol). However, the VdW and the electrostatic energies in this case (Congo Red and Glyconornicotine) differed only by 1.08 and 1.23 kcal/mol, respectively. Here, the torsional energy (bond angle) was the determining factor in the energy minimization. Similarly, in the case of Neocuproine, HMP, Apigenin, Thioflavin T, Curcumin and DHB, the energy differences were too close to determine their sensitivity and hence effectiveness against Aβ. Furthermore, even if only those NEs were considered which provided more stabilized structures than Aβ, 4 NEs will remain with Indomethacin very close to Aβ. Indomethacin, Curcumin, DHB and Glyconornicotine are less stabilized than Aβ in terms of Bond Length (bond energy) and torsional energy (angle). It is therefore extremely convoluted to isolate the most sensitive NEs and the most significant energy values for the inhibition of Aβ protein fibrillization or insolubilization of the plaque. To manage these issues machine learning approaches, such as Artificial Neural Networks (ANN) was employed for the selection of NEs with the most efficient/sensitive binding to Aβ protein.

### 2.2. Selection of Neuroprotective Entity (NE) Using Artificial Neural Networks

A maximum of 10,000 epochs were run on NeuroSolutions^®^ V5 (NeuroDimension Inc., Gainsville, FL, USA) for ensuring optimal training of data. Sensitivity analysis was used for extracting the cause and effect relationship between the inputs and the outputs of the network. This provided feedback pertaining to the input variable that was the most significant by testing the network with regard to its sensitivity about the binding to Aβ, thus elucidating the NEs that were most significant. Table 2 represents the input data in the form of energy attributes (obtained from the Molecular Mechanics simulations using AMBER Force Field) that was trained and the parameters used to construct the neural network are as shown in Table 3.

**Figure 3:**
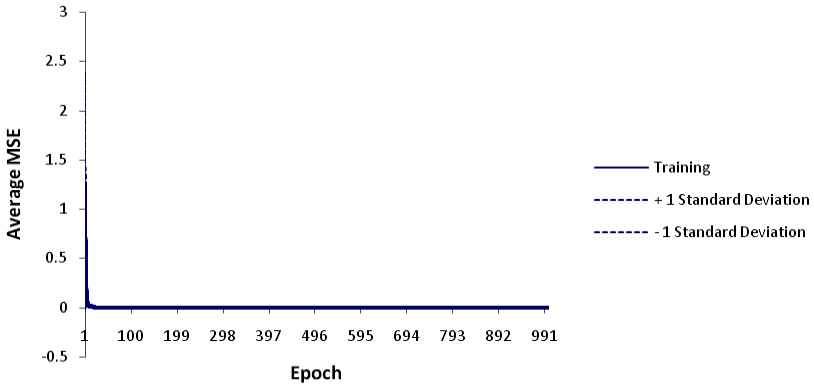
Average MSE for the ANN trains with standard deviations for 10,000 epochs.

For the 10 NEs employed in this study, the MLP network was able to accurately confirm that Curcumin and Glyconornicotine were the most significant NEs in terms of energy attributes of protein-ligand complexes based on the empirical data. The approach followed in this work required prior assumption for the selection of a mathematical model before applying the ANN models so as to be able to confirm the sensitivity coefficients of the various NEs as input variables that significantly contributed to characterizing the energy values. In order to obtain accuracy and maximum degree of precision, the training was undertaken twice (*i.e.*, primary and secondary training). The primary run (out of three runs) provided the lowest Mean Square Error (MSE) value. The leveling of the MSE with standard deviation (SD) boundaries for the training runs indicated highly improved data modeling as illustrated in Figure 3. Table 4 depicts the average of the MSE values for the three runs of the training, the best network run out of 10,000 epochs, and the overall efficiency and performance of the neural network during the data training.

**Figure 4:**
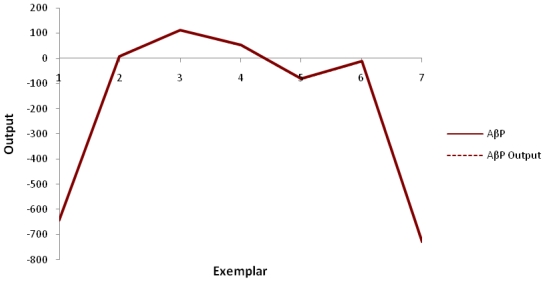
Mapping of correlation between desired and actual network output.

Basing on the results obtained, it was evident that the training model was highly efficient (MSE = 7.93561E-06). Results revealed a highly satisfactory fit for the input variables (*R*^2^ = 0.999). The performance criterion employed to assess the closeness and correlation between the desired and the actual network output for energy attributes of Aβ protein evidenced an extremely close mapping between the two outputs as illustrated in Figure 4.

**Figure 5:**
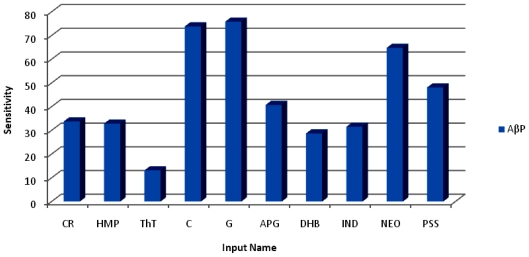
A typical bar chart showing the sensitivity coefficients of each NE against *AβP* following ANN sensitivity testing.

The sensitivity coefficient of each NE against the energy attributes of AβP is as shown in Figure 5. Glyconornicotine presented with the highest sensitivity against the energy attributes of AβP closely followed by Curcumin. This revealed their high capacity in stabilizing their respective protein-NE complexes. This behavior may be attributed to their high degree of non-bonding interaction in terms of Van der Waals forces, electrostatic interactions and H-bonding as shown in Table 1 and Figure 2. The sequence of the other remaining NEs in terms of sensitivity against AβP was NEO > PSS > APG > CR > HMP > IND > DHB >> ThT. The low sensitivity against AβP shown by DHB and IND despite forming highly stabilized protein-NE complexes (Table 1 and Figure 2) may be associated with higher torsional energy and lower Van der Waals forces. Results obtained from ANN sensitivity testing confirmed the relevance and efficiency of neural networks in optimization of ligand selection for effective Aβ protein targeting from an *in silico* modeling viewpoint.

### 2.3. Binding of Selected Neuroprotective Entities (NEs) to the Amyloid β-Protein

#### 2.3.1. Molecular Mechanics Simulations

**Figure 6:**
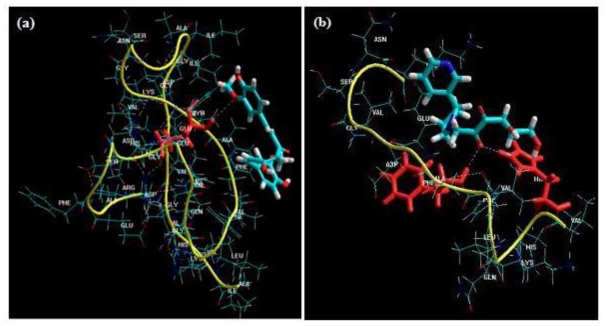
Visualization of binding of: (**a**) curcumin-glutamine acid [Aβ_25_]; and (**b**) glycosylated nornicotine- histidine and phenylalanine [Aβ_13_ & Aβ_20_] with Aβ protein. The NEs and the H-bonded residues (red) are depicted in tube rendering.

Static lattice atomistic simulations of protein-NE complexes for the ANN selected NEs (Curcumin and Glyconornicotine) exhibiting most sensitive Aβ protein binding was performed to explore the active sites for binding of the NEs. The minimizations were performed through the standard protocol described earlier in this paper. The H-bonds were recomputed after the AMBER Force Field computations as depicted in Figure 6. Curcumin, a phenolic yellow pigment with potent anti-inflammatory and antioxidant activities, has been reported to suppress oxidative damage, inflammation, cognitive deficits, and amyloid accumulation thereby affecting Aβ accumulation, oxidative damage, and inflammation, and other risks associated with Alzheimer‘s disease (AD) [8]. Additionally, the molecular structure of Curcumin has been investigated for its effects on Aβ aggregation whereby *in vitro* and *in vivo* studies demonstrated that Curcumin bound plaques, reduced amyloid levels and plaque burden, blocked aggregation and fibril formation as well as de-aggregation of Aβ [9]. In the present study, we modeled Curcumin molecule(s) in a close vicinity of Aβ, where it was found that Curcumin mostly formed H-bonds with the alanine residues present in the amyloid protein and were capable of binding to the aliphatic amino acids residues at various positions within the protein, mainly Aβ_12–28_ (Figure 6a). More than one molecule was also provided to interact with the protein structure as it has been proposed that the molar ratios for successful Aβ fibril and aggregate inhibition by Curcumin are greater than a 1:1 ratio [9].

On the other hand, the ability of nicotine to up-regulate the deficient nicotine receptors and covalently bind to helical Aβ structures hypothesized it as a neuroprotective agent in AD. Its metabolite nornicotine, and more specifically the glycosylated product formed by reaction of nornicotine with the ring-opened form of glucose to give the corresponding Amadori product, has been reported to cause aberrant nornicotine-based glycation of amyloid protein [12]. This 1,2-dicarbonyl-containing compound provides a reactive electrophile capable of covalently modifying protein residues by binding to aromatic residues such as phenylalanine and histidine (Figure 6). Therefore, this covalent chemical event may also preferentially alter the neurotoxicity of potentially toxic soluble aggregates thereby providing an intriguing and potentially valuable treatment for AD and other neurodegenerative disorders. Importantly, the ANN selected NEs, Curcumin and Glyconornicotine, were found to be interacting more specifically to the fragment VHHQKLVFFAEDVGSNK (Aβ_12–28_) which has been shown to be responsible for peptide aggregation in previous studies [9,12].

#### 2.3.2. Docking Studies

**Figure 7:**
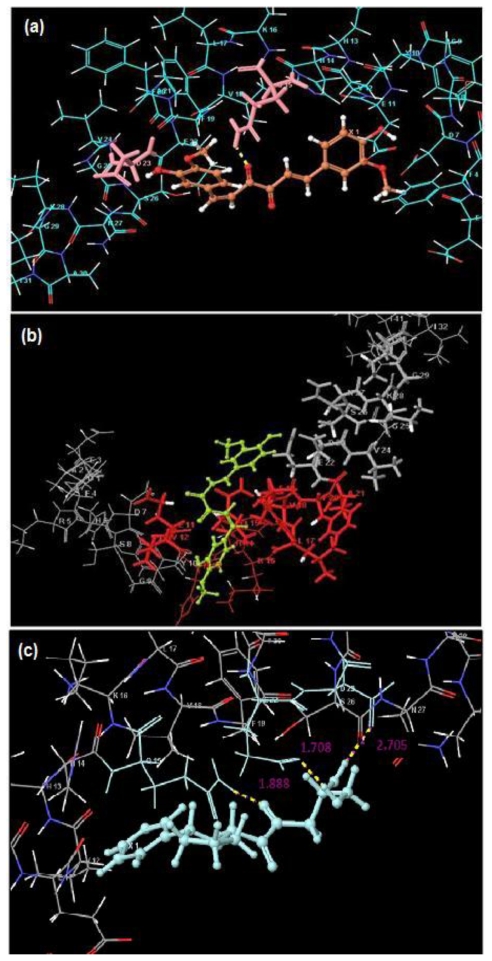
Binding of Curcumin (C) and Glyconornicotine (G) to respective sequences of the Aβ_1-42_ peptide with H-bond formation of the optimal poses docked into 1Z0Q also shown. (**a**) C: possibility of 2 H-bonds between the proteins (residues highlighted in pink); (**b**) C: highly stable pose apart from optimal conformation and (**c**) G: optimal pose with values (magenta indicates H-bonding distances).

To further analyze the molecular basis of interaction and affinity of binding of Curcumin and Glyconornicotine onto the Aβ_1-42_ peptide, the NEs were docked into the active site of the Aβ_1–42_ peptide. Docking results of these NEs are shown in Table 5. The ranking of NEs was based on the Glide score. Both NEs accepted poses with the protein (1Z0Q). The difference in Glide score between the NEs was minimal (±0.9) which revealed that the binding mode of both Curcumin and Glyconornicotine to Aβ_1-42_ may be considered similar. Results also demonstrated that docking simulations were able to dock both NEs at the fragment VHHQKLVFFAEDVGSNK (Aβ_12–28_) (Figure 7), even though they can form H-bonds at different sites as detailed later in this paper. Since Curcumin and Glyconornicotine have approximately the same sensitivity towards Aβ as revealed by ANN, it is therefore apparent that they may bind in a similar pattern to the active site of Aβ.

Upon comparison between the docking results of Curcumin and Glyconornicotine, the docking score was found to be superior in the case of Curcumin. However, Glynornicotine displayed sufficient interaction with the receptor especially in terms of H-bonding (which is considered an important factor in protein ligand interaction). In the case of Curcumin, 10 poses were generated, of which only four showed H-bonding interaction. On the contrary, in the case of Glyconornicotine, all 10 poses formed H-bonds. Figure 7 demonstrates the top-ranked poses with values indicating the H-bonding distances. Since all three distances are =/<2 Å this represents rather strong H-bonding interaction. The specific residue binding results of docking was consistent with the Molecular Mechanics simulation results as Glyconornicotine exhibited common H-bonding with phenylalanine in both the cases. This demonstrated the efficiency and accuracy of applied modeling methodologies in the present work for the alignment and generation of poses.

### 2.4. Isobolographic Analysis

The neuroprotective potential of Curcumin (C) and Glyconornicotine (G) was explored in a unique way involving the possibility of their synergistic action in terms of energy minimizations owing to different interaction sites as described by the docking studies. The potential of modeling Curcumin and Glyconornicotine in combination as a highly minimized energy of Aβ protein-NE complex in a synergistic manner was undertaken. The NEs, curcumin and glycosylated nornicotine, were modeled alone (2, 3 and 4 molecules in the case of Curcumin and 1, 2, 3, 4 and 5 molecules for Glyconornicotine) as well as in combination as fixed ratios of equi-effective energy responses for each NE. Several Molecular Mechanics investigations were performed to explore the individual effect of the NEs by modeling increasing number of molecules with the protein using AMBER Force Field (Table 6). The iso-effect (referred to as the minimum total energy achieved) was determined to be the highest response with the maximum number of NE molecules in the case of individual NEs *versus* the number of molecules in combination to produce the same effect (Table 6). Therefore, the total minimized energy achieved with four Curcumin molecules and five Glyconornicotine molecules was considered to be the iso-effect.

**Figure 8:**
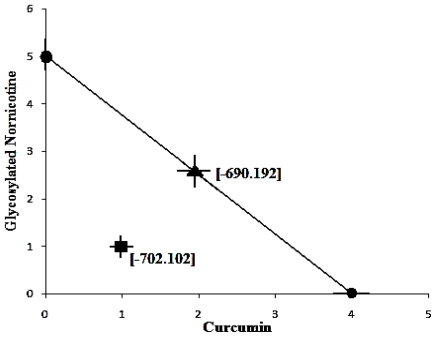
Isobologram showing the interaction between Curcumin and Glyconornicotine on the energetic response of Molecular Mechanics simulations. The number of molecules (for an energy minimized stable structure) of Curcumin and Glyconornicotine are contrived. The profile connecting the minimum energy points is the theoretical additive line, and the theoretical additive point (▴) for the NE combination is shown on the additive line. The minimized energy value (▪) of the combination of the two NEs was significantly lower than the theoretical additive value, indicating a synergistic interaction.

MM+ simulations were then performed using combinations such as C_1_-G_1_, C_1_-G_2_, C_2_-G_1_ and so forth to achieve the iso-effect energy level. Interestingly, the response obtained by AβP-(C)_1_-(G)_1_ was superior to that of four and five molecules of Curcumin and Glyconornicotine, respectively. Further, the isobologram of the combination of Curcumin and Glyconornicotine demonstrated that the experimentally derived energy values decreased below the theoretical molecule-additive limit, and the quantitative parameter (Λ) of the theoretical additive point and those of the experimental point did not overlap (Figure 8). This indicated a significant difference between the experimental and the theoretical additive point (*P <* 0.05) and a synergistic interaction between Curcumin and Glyconornicotine in the static lattice atomistic simulations. In addition, the total fraction value was 0.45, which was <1, indicating a synergistic interaction (Table 7).

### 2.5. Design of Experiments

It is fast becoming standard practice in research and development to employ Design of Experiments (DOE) methods, especially in the later stages of development, when the goal shifts from screening to product and process optimization [28]. Response Surface Methodology (RSM), such as the Central Composite Design, is the most popular class of RSM designs [29]. In this study a Face-Centered Central Composite Design (FCCCD) (Mintab^®^ V15, Minitab Inc., Boston, MA, USA) was employed with two study variables namely the number of Curcumin (C) and Glyconornicotine (G) molecules. The number of molecules of curcumin (X_1_) and glycosylated nornicotine (Glyconornicotine) (X_2_) was selected as the independent variables studied at two levels each (2–4 for curcumin and 1–5 for Glyconornicotine). Natural variable level settings for both molecules were used. The design consisted of four cube points, five center-points in cube, four axial points (points parallel to each variable axis on a circle of radius equal to 1.0 and origin at the center-point) and 0 axial center-points. An α = 1.0 defined a geometrically square design that was both rotatable and orthogonally blocked. Orthogonally blocked designs allow for model terms and block effects to be estimated independently and minimize the variation in the regression coefficients [30]. Rotatable designs provide the desirable property of constant prediction variance at all points that are equidistant from the design center, thus improving the quality of the prediction [31]. Since it was not be possible to have both properties for the FCCCD design that was selected, Minitab^®^ opted for orthogonal blocking and simultaneously attempted to converge as close as possible to the α value for rotatability. In order to ensure the successful optimization and prediction from the design, the region of operability encompassed the region of interest. The upper and lower limits of the independent variables were determined by modeling multiple NEs simultaneously. AβP-(C)_1_ and AβP-(C)_5_ were eliminated due to having higher total energy values as compared to and AβP and AβP-(C)_4_, respectively while AβP-(G)_6_ failed to converge even after 13125 cycles (Table 8). The number of Curcumin and Glyconornicotine molecules was in the region of interest described by the variable ranges. The design consisted of a two level full factorial with a total of 13 experimental runs.

Based on the orthogonal features of the design, a series of polynomial equations with one variable was obtained with the other six variables set at zero. Analysis of Variance (ANOVA), correlation analysis, path analysis, and regression analysis was used to analyze the dataset and statistical acceptability of the models proposed. The axial points and replicates were added to the design to provide for estimation of curvature of the models and to allow for estimation of experimental error.

#### 2.5.1. Analysis of the Face-Centered Central Composite Design

**Figure 9:**
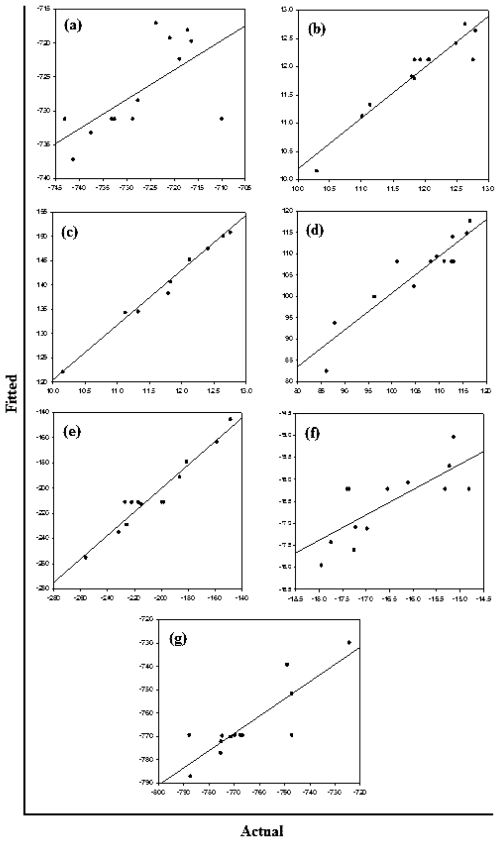
Linear correlation plots between fitted and actual values for (**a**) total steric energy; (**b**) bond length contributions; (**c**) bond angle contributions; (**d**) dihedral torsional contribution; (**e**) Van der Waals interactions; (**f**) ^h^ydrogen-bond energy function and (**g**) electrostatic energy.

The correlation of the independent variables and the responses were estimated by polynomial equations, using the least-square method. From a statistical point of view, three tests were used to evaluate the adequacy of the models; Student‘s *t*-test which is about the significance of factors, *R*-square test and Fisher tests. It was found that the individual effects were significant at a 5% significance level and only the interactions (CG), (CC), (GG) were not significant and were excluded during optimization. The test of reliability was performed by Fisher‘s variance ratio test known as the *F*-test. The tabulated *F* values at a 5% level of significance were between 1.08 and 12.42. Hence, it was concluded that the two variances are equal and that most of the response variation can be explained by the regression. Furthermore, the test for significance of regression confirmed that the established models provided an excellent fit to the observed data (Figure 9). Finally, the *R*^2^-value was found to be significantly high for the Bond Length, Bond Angle and Van der Waals forces with values of 89.9%, 89.0% and 92.8% respectively (Table 9). These variables were considered statistically relevant for both Curcumin and Glyconornicotine and therefore considered further in this study to proceed with optimization. In general, results also revealed that the difference between the measured and the fitted values did not exceed 3% indicating that the models can adequately represent the data. At a significance level of 0.05, the mean Curcumin and Glyconornicotine appeared not to be significant to the Total Energy (Table 9).

Estimated Regression Coefficients for all molecular attributes

(1) $$Total\;Energy=-731.182-1.951C+1.103G-4.005C^{2}+13.048G^{2}+3.263CG$$

(2) $$Bond\;Length=12.122+0.6593C+0.6423G-0.1377C^{2}-0.3519G^{2}-0.1760CG$$

(3) $$Bond\;Angle=145.220+7.771C+6.597G-2.946C^{2}-4.303G^{2}-1.512CG$$

(4) $$Dihedral=108.264+7.051C+10.566G-1.294C^{2}-3.981G^{2}-2.886CG$$

(5) $$VdW=-211.158-18.980C-35.792G+1.011C^{2}+11.950G^{2}-2.182CG$$

(6) $$H-bond=-16.2168+0.3307C+0.1756G+0.8547C^{2}-1.5644G^{2}+0.3488CG$$

(7) $$ES=-769.414+1.218C+18.915G-1.493C^{2}+11.298G^{2}+9.670CG$$

where, C = curcumin and G = glyconornicotine

The sequential and adjusted sums of squares (*i.e.*, Seq SS and Adj SS) (Table 3) were identical for all terms since the design matrix was orthogonal (Table 10).

**Figure 10:**
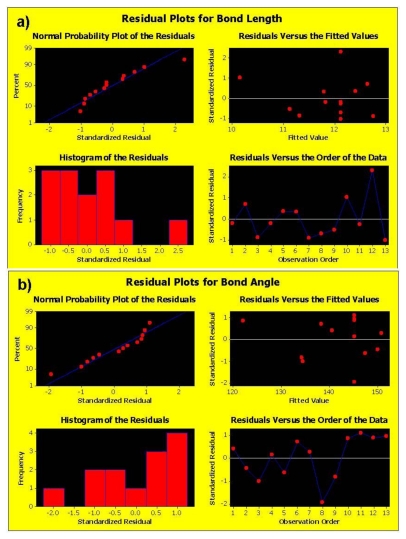

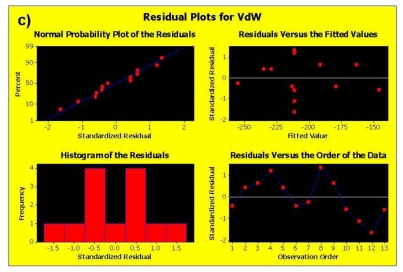
Linear correlation plots depicting corresponding residual plots for various variables.

**Figure 11:**
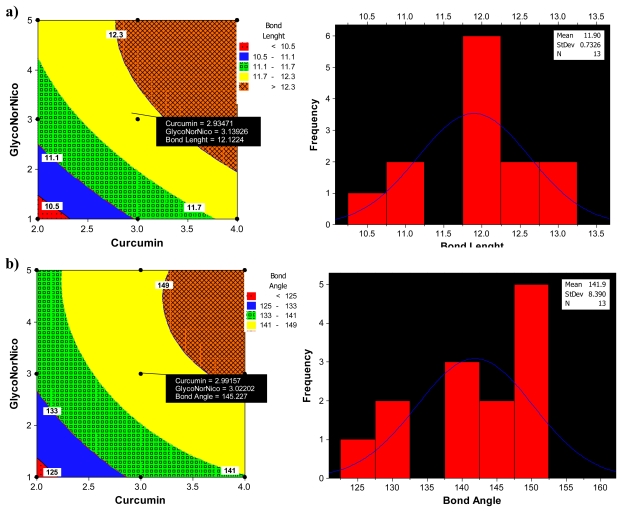

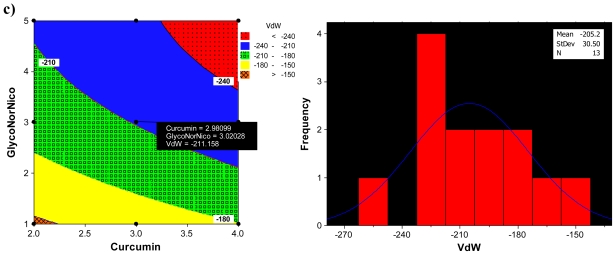
Corresponding Contour Plots of (**a**) bond length, (**b**) bond angle and (**c**) VdW *vs*. Curcumin and Glyconornicotine. The histograms show the frequency distribution of the 13 runs with respect to normality from the optimized value.

Figure 10a–c displays the diagnostic data for the design. The residuals *versus* fitted profiles displayed a large randomized spread in the data points for the highest fitted values. However, it is difficult to reject the assumption of constant variance in the residuals. The residuals for the variable Van de Waals (VdW) that was selected for optimization among the Bond Length and Bond Angle followed a relatively bell-shaped curve, though the Normal probability plot had two values off linearity at either end (corresponding to high and low values). This further established the significance of VdW in binding of Curcumin and Glyconornicotine to Aβ to afford neuroprotection. However, for the variables Bond Length and Bond Angle (Figures 10a,b) the *p-value* for the Anderson Darling test for Normality was >0.05 as well as the histograms displaying a bias in the frequency of the residuals below and above baseline. Hence the null hypothesis of Normality cannot be rejected and the mean of the residuals was zero. The I-chart (Individuals control chart) in the top right hand corner of Figures 10a–c assesses the independence assumption, and does not exhibit any concerning features. The variables Bond Length, Bond Angle and VdW for the NE combination were included in the statistical design for identifying the optimal NE combination and quantity of molecules required for neuroprotection. Figure 11a-c displays the 2D contour plots obtained for the three pertinent variables, Bond Length, Bond Angle and VdW employed for optimization. The optimized region (of 3 Curcumin:3 Glynornicotine) is also highlighted on the respective contour plots.

#### 2.5.2. Response Optimization

**Figure 12:**
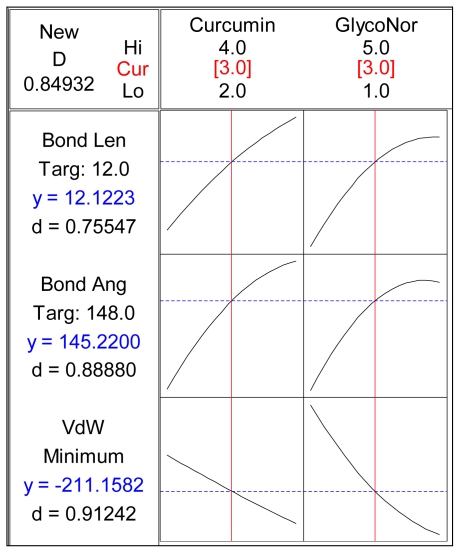
Desirability plots depicting the requisite variables for attributes of optimal combination with the desired targeted responses.

The Response Optimizer tool of Minitab^®^ V15 (Minitab Inc., Boston, MA, USA) was used to obtain the optimized levels of Curcumin and Glyconornicotine based on their molecular attributes in terms of Bond Length, Bond Angle and VdW. A single optimal combination was obtained following constrained optimization of the three variables as represented in Table 11. Upon comprehensive evaluation of feasibility searches and subsequently exhaustive grid searches, a neuroprotective combination of three molecules of Curcumin and three molecules of Glyconornicotine fulfilled the maximum requisites of an optimum combination primarily due to superior regulation of energy attributes. Figure 12 shows the desirability plots of each constraint for the optimized combination. Table 12 displays the local solution for Bond Length, Bond Angle and VdW in terms of the desirability score, predicted response, the actual response after Molecular Mechanics simulations of the optimized combination along with the percentage prediction errors. The prediction error for the response parameters ranged between 1.1 and 3.1% with the value of absolute error of 2.36. The low values of error indicated the high prognostic ability of the FCCCD employed in this study.

### 2.6. Discussion

**Figure 13:**
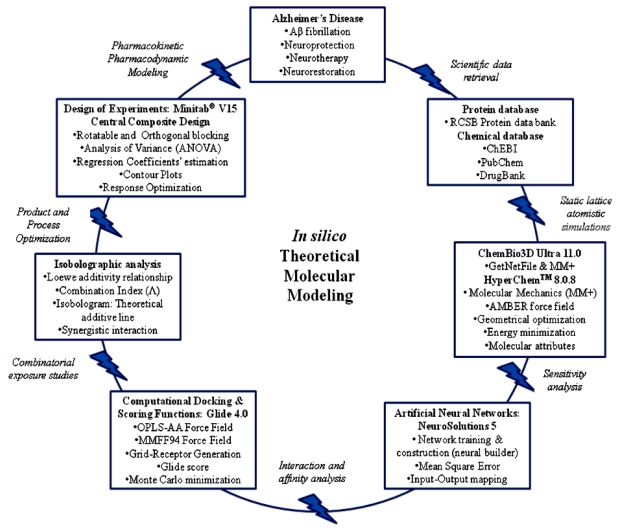
Schematic summarizing the *in silico* theoretical molecular model developed in this study for the screening of neuroprotective entities for Alzheimer’s disease.

This study provides a foremost comprehensive *in silico* evidence across combinatorial interventions for the potential neuroprotection and neurotherapy of Alzheimer’s disease (AD). *In silico* methods were developed to establish predictive models for concentration dependent interaction of neuroprotective entities (NEs) for modulating Aβ protein aggregation and oligomerization (Figure 13). Firstly, an extensive database search was performed to retrieve a library of small molecules, such as apigenin, congo red, curcumin, dihydroxybenzophenone, glycosylated nornicotine, hexadecylmethylpiperidinium, indomethacin, neocuproine, polystyrene sulfonate and thioflavin T, reportedly having high affinity for binding with Aβ protein leading to chemical disruption of the Aβ folding. Secondly, static lattice atomistic simulations, using ChemBio3D Ultra 11.0 and HyperChem™ 8.0.8, were performed throughout the study for quantifying the molecular attributes of the protein-ligand(s) interactions in the terms of various pertinent energy attributes and to generate preliminary data for protein-ligand sensitivity analysis, ligand-ligand interaction studies and combinatorial optimization. Thirdly, selection of NEs based on sensitivity analysis, employing ANN using NeuroSolutions^®^ V5, was conceded and the molecules Curcumin and Glycosylated nornicotine demonstrated higher sensitivities toward energy minimizations with Aβ based upon the Mean Square Error and input-output mapping.

Ligand selection was then followed by a detailed interaction studies using a more focused fragment-based geometrical optimization. This “binding surface hypothesis” was later substantiated through the use of docking studies (Glide 4.0). Previous studies have demonstrated the nicotine-based enhancement of memory function and reduction of cognitive deficits associated with experimental models of AD through multiple mechanisms, such as an increase in the expression of nicotinic acetylcholine (ACh) receptors, stimulating cortical ACh release and expression of cholinergic markers mediated by neurotrophic factors [32]. Additionally, an important mechanism involving the glycation of Aβ protein by glycosylated nornicotine recognized the chemical potential of this secondary metabolite to participate in potentially detrimental covalent chemistry leading to pathological consequences of nornicotine based protein glycation [12]. In our molecular modeling and docking studies, we explored the possible sites for glycation of Aβ within VHHQKLVFFAEDVGSNK (Aβ_12–28_) residues. Interestingly, the glucose side-chain of glycosylated nornicotine exhibited H-bonding with histidine and phenylalanine in the case of Molecular Mechanics simulations and with glutamine, phenylalanine and aspartic acid during the docking studies. Phenylalanine binding may potentially lead to glycation of the VFF tripeptide sequence of the Aβ protein which has been reported to be responsible for Aβ_12–28_-induced amnesia in a mouse model of Aβ toxicity [33]. Our *in silico* findings in terms of potential glycation sites, in addition to lysine reported by Dickerson and Janda (2003) [12], may lead to further exploration of the pathological consequences of nornicotine-based protein glycation. On the other hand, curcumin delivery has been reported to have pleiotropic activities relevant to AD including stimulation of phagocytic Aβ clearance, anti-inflammatory and antioxidant activity, metal chelation, neurogenesis and Aβ-and Tau-binding properties [9,34]. In the present study, we modeled the Aβ-binding properties of Curcumin for the exploration of possible interaction sites. The binding outcome varied in both the case as Curcumin exhibited binding with glutamine and glutamic acid in case of Molecular Mechanics simulations and docking studies respectively. Additionally, Curcumin demonstrated binding to the ends of the full Aβ (data not shown).

Once the different binding sites were confirmed, a Curcumin and Glyconornicotine interaction analysis was performed for synergism, if any. A highly synergistic interaction was observed displaying a possible reduction in individual effective concentration by a factor of 4 and 5, respectively, without compromising and even substantiating the therapeutic benefit. This reduction in concentration levels may have implications in overcoming the notions related to nicotine-addiction and low brain uptake of Curcumin. Finally, combinatory optimization in terms of requisite variables and maximum-stabilization with the desired targeted responses was conducted employing Design of Experiments using Minitab^®^ V15. A neuroprotective combination of three molecules of Curcumin and three molecules of Glyconornicotine was proposed by the model indicating a possible 1:1 combination with maximum of three molecules of each NE per Aβ oligomer. Thus, our work offers a mathematical and *in silico* approach that constitutes a new frontier in providing neuroscientists with a template for *in vitro* and *in vivo* molecular experimentation. Future work is recommended in terms of *in vivo* pharmacokinetic and pharmacodynamic modeling for the verification of the above theoretical modeling.

## 3. Experimental Section

### 3.1. Preparation of Protein Target Structure and Compound Libraries

**Figure 14:**
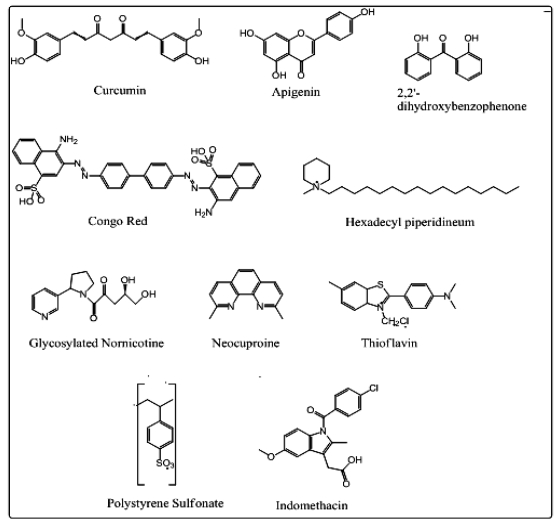
Neuroprotective Entities (NEs) employed in this study for modeling simulations.

The starting coordinates of the Alzheimer’s disease (AD) amyloid β(1–42) peptide (Aβ_1–42_) [PBD ID: 1Z0Q] and AD amyloid β(1–42) fibrils [PBD ID: 2BEG] were obtained from the Protein Data Bank (www.pdb.org) [35] and further modified for molecular mechanics and docking computations. The coordinates of ligand Neuroprotective Entities (NEs) (Figure 14) were obtained from the ChEBI database (Chemical Entities of Biological Interest) (www.ebi.ac.uk/chebi*)* [36] and included the following: apigenin (ChEBI: 18388), congo red (ChEBI: 34653), curcumin (ChEBI: 3962), indomethacin (ChEBI: 49662) and polystyrene sulfonate (PSS) [ChEBI: 53280]. Inhibitor derivatives such as dihydroxybenzophenone (DHB), hexadecyl-*N*-methylpiperidinium (HMP) and glycosylated nornicotine (GlycoNorNicotine) were built using benzophenone (ChEBI: 41308), piperidinium ion [ChEBI: 48633] and nornicotine [ChEBI: 28313] as templates, respectively. Neocuproine and Thioflavin T (ThT) (PubChem: 16953) were selected from The Timely Data Resources (TDR) Targets Database (www.tdrtargets.org) [37] and PubChem (www.pubchem.ncbi.nlm.nih.gov) [38].

### 3.2. Static Lattice Atomistic Simulations

Molecular Mechanic Computations in vacuum were performed using HyperChem™ 8.0.8 Molecular Modeling Software (Hypercube Inc., Gainesville, FL, USA) and ChemBio3D Ultra 11.0 (CambridgeSoft Corporation, Cambridge, UK). The Aβ_1–42_ peptide molecule and Aβ_1–42_ fibril was downloaded using the GetNetFile Tool in ChemBio3D Ultra in their syndiotactic stereochemistry as 3D models and saved in an appropriate HyperChem™ compatible file format for further processing and computations. The structure of Aβ_12–28_ peptide chain was generated using the Sequence Editor Module on HyperChem™. Structures of various Aβ inhibitor ligand Neuroprotective Entities (NEs) were constructed employing innate bond angles as defined in Hyperchem™. The models were initially energy-minimized using MM+ Force Field and the resulting structures were once more energy-minimized using the AMBER (Assisted Model Building and Energy Refinements) Force Field. The conformer having the lowest energy was used to create the target-ligand complexes. A complex of one molecule with another was assembled by parallel disposition, and the same procedure of energy-minimization was repeated to generate the final models constituting: AβP-CR (congo red), AβP-HMP (hexadecyl-*N*-methylpiperidinium), AβP-ThT (thioflavin T), AβP-C (curcumin), AβP-G (glyconornicotine), AβP-APG (apigenin), AβP-DHB (dihydroxybenzophenone), AβP-IND (indomethacin), AβP-NEO (neocuproine), AβP-PSS (polystyrene sulfonate) (for selection of the most sensitive Aβ-inhibitor/neuroprotective entity using Artificial Neural Networks sensitivity testing) and curcumin-AβP-glyconornicotine molecules at varying concentrations (for optimization of the highly minimized ternary complex using a Face-Centered Central Composite Design). Full geometrical optimizations were performed in vacuum employing the Polak–Ribiere conjugate gradient method until a Root Mean Square (RSM) gradient of 0.001 kcal/mol was reached. Force Field options in the AMBER (with all hydrogen atoms explicitly included) and MM+ (extended to incorporate non-bonded restraints) methods were set at HyperChem™ user defaults. For Molecular Mechanics computations, the Force Fields were utilized with a distance-dependent dielectric constant scaled by a factor of 1. The 1–4 scale factors were as follows: electrostatic 0.5 and van der Waals 0.5. Furthermore, various energies and molecular attributes involved in the molecular interactions were computed.

### 3.3. Sensitivity Testing by Artificial Neural Networks for Optimal Neuroprotective Entity Selection

**Figure 15:**
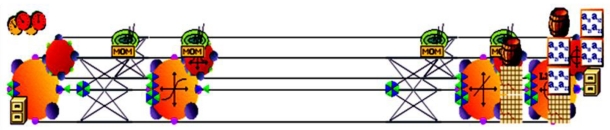
Schematics depicting the **c**onstructed Multilayer Perceptron network and the network topology for the hidden input and output layers employing Artificial Neural Networks.

Sensitivity testing and optimization was conducted by employing a feedback Multilayer Perceptron (MLP) neural network to train the empirical input bond-energy data with static back propagation (NeuroSolutions^®^ V5, FL, USA). The MLP is a layered feedforward network typically trained with back propagation of errors using gradient descent or conjugate gradient algorithms. The advantage of being able to approximate any input/output map makes an MLP highly useful in applications requiring static pattern classification [39]. Figure 15 illustrates the typical MLP network constructed and the network topology for the hidden input and output layers. A genetic algorithm with a Sigmoid Axon transfer function and Conjugated Gradient learning rule was employed for the hidden input and output layers.

### 3.4. Prediction of the Structure and Binding Affinity of Target-Ligand Complexes

For preparation of the protein target structure, the Aβ complex obtained from the Protein Data Bank was modified for docking computations via Glide 4.0 software (Schrödinger LLC, New York, NY, USA, 2005). The computations were performed by importing the Aβ complex to Maestro (Schrödinger) along with identifying and eliminating co-crystallized ligands and further minimized using the Protein Preparation wizard applying an OPLS-AA Force Field (autoref.pl script). Minimizations were performed until the average Root Mean Square (RSM) deviation of the non-hydrogen atoms reached 0.3 Å [40]. The ligand (obtained from databases as described in the materials section) analogue library was generated by modifying the respective functional groups using the reagent database and a combinatorial design module. Each structure was assigned an appropriate bond order (LigPrep script). The inhibitors were converted to Mae format (Maestro, Schrodinger, Inc.) and optimized by means of the MMFF94 Force Field using user defaults [41]. Glide 4.0 computations such as docking and scoring functions were performed with various scaling factors for the Van der Waal radii of the receptor and ligand atom. The receptor-grid files were generated using a Grid- Receptor Generation algorithm after ensuring that the protein and ligands were in the correct form for docking. The size of ligands to be docked was selected from the workspace and was docked with the active site using the ‘Xtra Precision’ algorithm. Conformations were generated internally and conceded these through a series of filters involving Grid-Based Force Field evaluation and refinement of docking solutions including torsional and rigid body motions of the ligand using the OPLS-AA Force Field. The surviving docking solutions were then subjected to Monte Carlo procedure minimization of energy scores and the final energy evaluations were performed with GlideScore to generate the single best pose as the output for a particular ligand.

### 3.5. Interaction Studies Employing Isobolographic Analysis

The Loewe additivity relationship was used to analyze interactions between each Neuroprotective Entity (NE). The equation assumes that the fractional effect contributed from each NE is additive to explicate the entire response from combinations. The Combination Index (Λ) is calculated using Equation (8).

(8) $$\Lambda =\frac{C_{1}}{C_{i*}}+\frac{C_{2}}{C_{2*}}+\ldots +\frac{C_{n}}{C_{n*}}$$

where, C_i_ are the concentrations of various NEs in combination, and C_1_* are the concentrations of various NEs that would produce the same effect when used alone. A “Λ” of <, =, or > 1 indicates synergy, additivity, and antagonism, respectively [42]. Isobolographic analysis for the combination of most sensitive NEs (as determined by ANN), in terms of contribution to the energy minimization of target-ligand complex, was conducted based on comparisons of a number of interacting ligand molecules that were determined to be equi-effective. The NEs were modeled alone as well as in combination as fixed ratios of equi-effective energy responses for each NE. The energy minimized confirmations of the combined NEs were used to compute the various pertinent energies and molecular attributes involved in the molecular interactions. The isobolos were constructed by plotting the Total Energy values of one NE on the independent axis and that of other as the dependent variable, modeled alone and in combination.

### 3.6. Design of Experiments

A Face-Centered Central Composite Design (FCCCD) with α = 1 was employed as per standard protocol. The number of molecules of NEs, X_1_ and X_2_, were selected as the independent variables studied at two levels each (2–4 for curcumin and 1–5 for glyconornicotine). The upper and lower limits were determined by performing MM+ simulations of protein-NE complexes as shown in Table 6. The central point (0, 0) was studied in quintuplicate. All other processing variables were kept invariant throughout the study. Table 8 summarizes an account of the 13 experimental runs studied, their factor combinations and the responses obtained after subsequent modeling simulations undertaken. The Total Energy, Bond Length, Bond Angle, Dihedral, Van der Waals, H-Bonding and Electrostatic energies were specified as the response variables. Various Response Surface Methodology computations for the current optimization study were performed employing Minitab^®^ V15 software (Minitab Inc., Boston, MA, USA). Polynomial models including interaction and quadratic terms were generated for the response variables using Multiple Linear Regression Analysis (MLRA). The general form of the model is represented in Equation (9).

(9) $$Y=\beta_{0}+\beta_{1}X_{1}+\beta_{2}X_{2}+\beta_{3}X_{1}X_{2}+\beta_{4}{X_{1}}^{2}+\beta_{5}{X_{2}}^{2}+\beta_{6}X_{1}{X_{2}}^{2}+\beta_{7}{X_{1}}^{2}X_{2}$$

where, β_0_ is the intercept representing the arithmetic average of all quantitative outcomes of 13 runs; β_1_ to β_7_ are the coefficients computed from the observed experimental values of Y; and X_1_ and X_2_ are the coded levels of the independent variable(s). The terms X_1_X_2_ and Xi^2^ (i = 1 to 2) represent the interaction and quadratic terms, respectively. Statistical validity of the polynomials was established on the basis of ANOVA provision in the Minitab^®^ V15 software. Subsequently, feasibility and grid searches were performed to locate the composition of the optimum combinations (*i.e.*, optimum number of NE molecules to ensure Aβ binding and neuroprotection). In addition, 2D contour plots were constructed using the design outputs generated in order to visualize the data regions of interest. Numerical optimization using the desirability approach was employed to locate the optimal settings of the independent variables in order to obtain the desired response. An optimized formulation was developed by setting constraints on the dependent and independent variables. The optimized combination developed was evaluated for the responses and the experimental values obtained were compared with those predicted by the mathematical models generated.

## 4. Conclusions

Our approach was to assay a library of potential neuroprotective entities (NEs) that were previously reported to address binding features that are critical for inhibition of Aβ aggregation. Our findings indicate that a combination of NEs may result in synergistic activity with a significant reduction in dose. Firstly, in the ANN sensitivity testing, we selected curcumin and glyconornicotine as the most sensitive NEs toward stabilizing the Aβ protein. It is worth noting that, both these compounds have been shown to inhibit Aβ aggregation by other experimental approaches [9,12]. In the second component of our study, we used isobolographic analysis simultaneously with Design of Experiments to deduce the interrelation between both the NEs in terms of synergism and their collective influence on the Aβ binding at concentration rations of ligand:ligand, 1:1, and protein:ligand, 1:3. In addition, the molecular basis of interaction and affinity of binding of curcumin and glyconornicotine onto the Aβ_1–42_ peptide was deduced using Molecular Mechanics and Advanced Docking computations. Results obtained from *in silico* from this study suggest that curcumin and glycosylated nornicotine can form a potential neuroprotective and neurotherapeutic combination against aggregated Aβ that causes Alzheimer’s disease. We anticipate that in future neuroscientists would adopt this *in silico* approach to develop novel therapeutic interventions for the neuroprotection and neurotherapy of Alzheimer’s disease or as a template for other therapeutic strategies. In addition, the neuroprotective entities identified in this study may also be valuable in this regard.

## Figures and Tables

**Table 1:** Computed energy parameters (kcal/mol) of the protein-NE complexes.

Molecule	Total Energy^a^	Bond Length^b^	Bond Angle^c^	Dihedral^d^	VdW^e^	H-bond^f^	ES^g^
**AβP**	−642.58	8.86	112.07	54.04	−80.42	−11.19	−728.89
**AβP-CR**	−596.30	10.97	174.75	74.36	−103.44	−18.33	−734.44
**AβP-PSS**	−606.71	9.51	171.42	65.70	−102.03	−18.17	−733.17
**AβP-NEO**	−618.54	8.28	107.03	51.66	−83.07	−16.86	−686.59
**AβP-HMP**	−619.63	8.34	103.03	50.15	−89.66	−12.64	−677.26
**AβP-APG**	−636.01	8.34	108.51	49.90	−89.94	−17.39	−692.48
**AβP-ThT**	−638.33	10.51	135.38	67.69	−108.33	−16.17	−727.29
**AβP-IND**	−643.98	9.51	128.76	65.28	−99.51	−17.97	−730.10
**AβP-C**	−652.31	9.63	121.25	68.97	−102.44	−18.38	−731.38
**AβP-DHB**	−653.94	9.42	114.75	64.71	−94.19	−18.50	−730.18
**AβP-G**	−666.07	9.15	119.25	61.92	−104.52	−18.62	−733.21

a Total steric energy for an optimized structure

b Bond stretching contributions, reference values were assigned to all of a structure's bond lengths

c Bond angle contributions, reference values were assigned to all of a structure's bond angles

d Torsional contribution arising from deviations from optimum dihedral angles

e van der Waals interactions due to non-bonded interatomic distances

f Hydrogen-bond energy function

g Electrostatic energy

**Table 2:** Input Data for neural network training, computations and sensitivity testing.

Attribute	CR^a^	HMP^b^	ThT^c^	C^d^	G^e^	APG^f^	DHB^g^	IND^h^	NEO^i^	PSS^j^	AβP^k^
**Total Energy**	−596.30	−619.63	−638.33	−652.31	−666.07	−636.01	−653.94	−643.98	−618.54	−606.71	−642.58
**Bond Length**	10.79	8.37	10.35	9.64	9.13	8.39	9.48	9.55	8.29	9.53	8.81
**Bond Angle**	174.75	103.03	135.38	121.25	119.25	108.51	114.75	128.76	107.03	171.42	112.07
**Dihedral**	74.36	50.15	67.69	68.97	61.920	49.90	64.71	65.28	51.66	65.70	54.04
**VdW**	−103.44	−89.66	−108.30	−102.42	−104.55	−89.94	−94.19	−99.51	−83.07	−102.03	−80.42
**H-bond**	−18.33	−12.64	−16.17	−18.38	−18.627	−17.39	−18.50	−17.97	−16.86	−18.17	−11.19
**ES**	−734.44	−677.26	−727.29	−731.38	−733.21	−692.48	−730.18	−730.10	−686.59	−733.17	−728.89

a Congo red;

b Hexadecylmethylpiperidinium;

c Thioflavin T;

d Curcumin;

e Glycosylated nornicotine;

f Apigenin;

g Dihydroxybenzophenone;

h Indomethacin;

I Neocuproine;

j Polystyrene sulfonate;

k Amyloid-β Protein.

**Table 3:** Artificial Neural Networks construction parameters employing a neural builder.

Parameter	Setting
Hidden layer	1
Exemplars	17
Output Processing element	1
Transfer function	SigmoidAxon:sigmoid (0/1)
Learning rule	ConjugateGradient: second order method for gradient
Maximum Epochs	10,000: Supervised Learning Control Termination at Mean Square Error; Load Best Weights Approach
Probe Configuration	Quantitative-MatrixViewer, MatrixEditor; Qualitative-MegaScope, Hinton

**Table 4:** Neural network indicators characterizing the efficiency and performance of data in the training as per ANN.

Best Network	Training	Performance	*AβP*
Epoch #	10,000	MSE	3.457756864
Minimum MSE^a^	7.93561E-06	NMSE^b^	3.31882E-05
Final MSE	7.93561E-06	MAE^c^	1.749398046
-	-	Min Abs Error^d^	0.887866082
-	-	Max Abs Error^e^	2.866526507
*R^2^*	-	-	0.999983413

a MSE: Mean square error

b NMSE: Normalized mean square error

c MAE: Mean absolute error

d Min Abs Error: Minimum absolute error

e Max Abs Error: Maximum absolute error

**Table 5:** Docking results of curcumin and glyconornicotine in the original crystal structure of amyloid β protein (1Z0Q) using Glide-xp.

Rank	Ligand	No. of poses generated	Glide score	*Δ*Score	H-bond^a^ length (A˚)	Emodel^b^ (kcal/mol)
1	Curcumin	10	−3.79	−0.9_(1–2)_	2.73	−37.3
2	Glycosylated nornicotine	10	−2.89	+0.9_(2–1)_	1.89	−33.3

a Average of all bond lengths

b Emodel is a specific combination of GScore, CvdW and the internal torsional energy of the ligand conformer.

**Table 6:** Computed energy parameters (kcal/mol) of the protein-NE complexes.

Molecule	Total Energy^d^	Bond Length^e^	Bond Angle^f^	Dihedral^g^	Vdw^h^	H-bond^i^	ES^j^
**AβP**	−642.583	8.816	112.076	54.040	−80.426	−11.192	−728.898
**AβP-(C)1**^a^	−582.939	11.447	179.994	80.810	−104.533	−17.946	−732.712
**AβP-(C)2**	−652.313	9.643	121.259	68.974	−102.424	−18.382	−731.383
**AβP-(C)3**	−660.406	11.318	139.298	104.718	−138.832	−18.819	−757.09
**AβP-(C)4**	−682.039	12.910	150.151	115.683	−164.561	−17.052	−779.169
**AβP-(C)5**	−676.414	13.833	158.07	127.859	−179.578	−17.251	−779.348
**AβP-(G)1**^b^	−666.074	9.135	119.259	61.920	−104.552	−18.627	−733.21
**AβP-(G)2**	−673.763	9.402	122.92	66.095	−120.558	−18.899	−732.723
**AβP-(G)3**	−681.320	9.718	123.795	70.168	−137.011	−19.282	−728.71
**AβP-(G)4**	−691.403	10.076	129.734	71.880	−151.861	−20.955	−730.279
**AβP-(G)5**	−698.344	10.536	132.023	77.219	−169.188	−21.046	−727.889
**AβP-(G)6**	NOT Converged (13125 cycles 28811 points)						
**AβP-(C)1-(G)1**^c^	−702.102	9.752	121.608	80.58	−127.923	−17.01	−769.11

a AβP-(C)_n_: n is the number of molecule(s) of curcumin.

b AβP-(G)_n_: n is the number of molecule(s) of glyconornicotine.

c AβP-(C)_n_-(G)_n_: n is the number of molecule(s) of curcumin and glyconornicotine.

d Total steric energy for an optimized structure.

e Bond stretching contributions, reference values were assigned to all of a structure’s bond lengths.

f Bond angle contributions, reference values were assigned to all of a structure’s bond angles.

g Torsional contribution arising from deviations from optimum dihedral angles.

h van der Waals interactions due to non-bonded interatomic distances.

i Hydrogen-bond energy function.

j Electrostatic energy.

**Table 7:** Quantitative parameter (Λ) determination for interaction between curcumin and glycosylated nornicotine.

Molecule	Response (units)
^a^**AβP-(Curcumin)4**	−682.039
^b^**AβP-(GlycoNorNicotine)5**	−698.344
Isoeffect*	−690.192
^c^**AβP-(Curcumin)1-**^d^**(GlycoNorNicotine)1**	−702.102
Quantitative parameter (Λ)**	0.45

* Average of AβP-(Curcumin)_4_ and AβP-(GlycoNorNicotine)_5_

** $\Lambda =\frac{c}{a}+\frac{d}{b}=\frac{1}{4}+\frac{1}{5}=0.45$

**Table 8:** Randomized Face-Centered Central Composite Experimental Design Template.

F#	C^a^	G^b^	Total Energy^c^	Bond Length^d^	Bond Angle^e^	Dihedral^f^	Vdw^g^	Hbond^h^	ES^i^
**1**	4	1	−727.66	11.79	141.36	104.69	−181.21	−16.98	−787.32
**2**	4	3	−741.28	12.79	148.87	112.83	−225.90	−15.13	−774.75
**3**	2	3	−737.52	11.13	131.88	96.25	−186.38	−15.23	−775.20
**4**	3	3	−733.11	12.06	145.71	111.11	−199.46	−14.81	−787.73
**5**	3	5	−723.80	12.49	145.85	115.90	−231.79	−17.25	−748.99
**6**	2	5	−718.92	11.83	139.50	109.48	−214.81	−17.74	−747.17
**7**	4	5	−716.37	12.63	151.29	116.57	−256.36	−16.10	−724.40
**8**	3	3	−710.02	11.92	138.78	101.08	−198.06	−16.54	−747.21
**9**	3	1	−720.89	11.01	132.18	87.816	−158.60	−17.95	−775.35
**10**	2	1	−717.16	10.29	123.51	86.057	−148.40	−17.22	−771.40
**11**	3	3	−732.58	12.05	148.88	112.68	−222.08	−17.40	−766.71
**12**	3	3	−743.03	12.76	148.15	108.23	−227.16	−15.31	−769.70
**13**	3	3	−728.71	11.84	148.35	113.06	−217.03	−17.36	−767.57

a Curcumin

b Glyconornicotine

c Total steric energy for an optimized structure

d Bond stretching contributions, reference values were assigned to all of a structure’s bond lengths

e Bond angle contributions, reference values were assigned to all of a structure’s bond angles

f Torsional contribution arising from deviations from optimum dihedral angles

g van der Waals interactions due to non-bonded interatomic distances

h Hydrogen-bond energy function

i Electrostatic energy

**Table 9:** Pertinent statistical descriptors for determining the model adequacy (*p-values* and *R^2^*).

	C^a^	G^b^	C^2^^c^	G^2^^d^	CG^e^	R^2^
**Total Energy**	0.649	0.796	0.529	0.068	0.537	43.5
**Bond Length**	0.001*	0.001*	0.478	0.097	0.287	89.9
**Bond Angle**	0.001*	0.003*	0.221	0.091	0.435	89.0
**Dihedral**	0.010	0.001*	0.678	0.224	0.283	86.1
***VdW***	0.003*	0.000*	0.880	0.106	0.696	92.8
**H-bond**	0.414	0.659	0.172	0.027	0.479	57.6
**ES**	0.812	0.006*	0.843	0.164	0.154	74.0

a Curcumin

b GlycoNorNicotine

c Curcumin*Curcumin

d GlycoNorNicotine*GlycoNorNicotine

e Curcumin*GlycoNorNicotine

* indicates statistically significant values

**Table 10:** Analysis of Variance for all molecular attributes.

	Total Energy	Bond Length	Bond Angle	Dihedral	VdW	H-bond	ES
aSeq SS	545.95	5.7884	751.627	1070.68	10361.38	8.3031	2907.70
bAdj SS	545.95	5.7884	751.627	1070.68	10361.38	8.3031	2907.70
cP-value	0.447	0.002*	0.003*	0.006*	0.001*	0.212	0.050
dF	1.08	12.42	11.30	8.69	18.09	1.90	3.98

a Sequential Sum of Squares (Source: Regression)

b Adjusted Sum of Squares (Source: Regression)

c P-value (Source: Regression)

d F (Source: Regression)

**Table 11:** Variable constraints employed for response optimization.

Parameters	Goal	Lower	Target	Upper	Weight
**Bond Length**	Target	10	12	12.5	1
**Bond Angle**	Target	123	148	148.5	1
**VdW**	Minimum	−256	−256	−1.00	1

**Table 12:** Actual and predicted response values for the optimized formulation.

Local Solution	Desirability	Predicted Responses	Actual Responses	Error (%)
**Bond Length**	0.75547	12.1223	12.4932	2.9
**Bond Angle**	0.88880	145.2200	149.7621	3.1
**VdW**	0.91242	−211.1582	−213.5112	1.1

**Absolute error = 2.36**				
